# Factors associated with the desire to quit tobacco smoking in Saudi Arabia: Evidence from the 2019 Global Adult Tobacco Survey

**DOI:** 10.18332/tid/159735

**Published:** 2023-03-03

**Authors:** Sarah S. Monshi, Turky J. Arbaein, Abdulrhman A. Alzhrani, Ali M. Alzahrani, Khulud K. Alharbi, Afrah Alfahmi, Mansour Z. Alqahtani, Ali H. Alzahrani, Ahmed A. Elkhobby, Aljoharah M. Almazrou, Abdullah M. M. Alanazi

**Affiliations:** 1 Department of Health Services Management, College of Public Health and Health Informatics, Umm Al-Qura University, Makkah, Saudi Arabia; 2 Tobacco Control Program, Ministry of Health, Riyadh, Saudi Arabia; 3 Department of Respiratory Therapy, College of Applied Medical Sciences, King Saud bin Abdulaziz University for Health Sciences, Riyadh, Saudi Arabia; 4 King Abdullah International Medical Research Center, Riyadh, Saudi Arabia

**Keywords:** smoking cessation, tobacco control, desire to quit, Saudi smokers

## Abstract

**INTRODUCTION:**

Saudi Arabia is expected to witness a slight reduction in tobacco use. The Saudi government offers free-of-charge smoking cessation services. Yet, factors influencing the desire to quit smoking are not comprehensively investigated in Saudi Arabia. This study examines the factors influencing the desire to quit among smoking adults in Saudi Arabia and investigates whether using alternative tobacco products, such as e-cigarettes, is associated with the desire to quit smoking.

**METHODS:**

Data from the 2019 nationally representative Global Adults Tobacco Survey (GATS) was used. GATS utilized a face-to-face household cross-sectional survey that collected data from adults aged ≥15 years. Several factors including, sociodemographic characteristics, use of alternative tobacco products, attitude toward tobacco control, and awareness of smoking cessation clinics (SCCs), were examined to predict the desire to quit. Logistic regression analysis was conducted.

**RESULTS:**

A total of 11381 individuals completed the survey. Of the total sample, 1667 participants were tobacco smokers. The majority of the tobacco smokers were interested in quitting smoking (82.4%); 58% of cigarette smokers and 17.1% of waterpipe smokers were interested in quitting smoking. The desire to quit smoking was positively associated with the awareness of SCCs (AOR=3; 95% CI: 1.8–5), attitude toward raising tobacco taxes (AOR=2.3; 95% CI: 1.4–3.8), and a strict rule of smoking inside the home (AOR=2; 95% CI: 1.1–3.9). No statistical association was found between the desire to quit smoking and the use of e-cigarettes.

**CONCLUSIONS:**

The desire to quit tobacco smoking among Saudi smokers increased with awareness of SCCs, favoring taxes on tobacco products, and implementing strict rules of smoking inside the home. The study reveals valuable insights into the main factors that could inform the development of more effective policy interventions targeting smokers in Saudi Arabia.

## INTRODUCTION

Tobacco smoking is a significant public health concern and a leading cause of preventable death worldwide. Direct and indirect tobacco smoking kills more than eight million people annually^1^. It is a significant risk factor for many chronic diseases, such as cancers, chronic obstructive pulmonary disease (COPD), and cardiovascular diseases^2^. According to the World Health Organization (WHO), by 2025, tobacco control initiatives are anticipated to have reduced tobacco prevalence rates throughout all WHO regions^3^. The recent assessment of epidemic smoking over the past half century shows a reduction in the overall smoking prevalence among adult males (32.2% to 33.1%) and females (6.3% to 6.7%)^4^. However, the reduction in tobacco use is unequal across countries^4^. The Eastern Mediterranean region, including Saudi Arabia, is one of the regions that is expected to witness the smallest reduction^3^.

In Saudi Arabia, many people die each year from smoking cigarettes^2^. The prevalence of tobacco use among adults in the country is 19.8% (30% among men and 4.2% among women)^5^. Furthermore, the direct and indirect costs of smoking and secondhand smoke exposure in Saudi Arabia were the highest among the six Gulf Cooperation Council (GCC) countries in 2016, at a total of $17.2 billion^6^. There are several patterns of tobacco use, such as conventional cigarettes, smokeless products, and waterpipe (also called hookah or shisha)^5^. Emerging tobacco products have also occurred in Saudi Arabia, including heated tobacco products (HTPs) and electronic cigarettes (e-cigarettes)^5^. Waterpipe and e-cigarettes are more common among women and youth, since they are more appealing than conventional cigarettes, and both tobacco products are associated with a misconception about their harmful effects^7-9^.

The Saudi government has made significant efforts to address the issue of tobacco use. In 2005, Saudi Arabia ratified the WHO Framework Convention on Tobacco Control (FCTC) treaty^10^. The FCTC consists of tobacco control policies targeting the demand and supply of tobacco to help nations overcome the issue of tobacco use^10^. Later, in 2015, Saudi Arabia introduced the anti-smoking law to combat tobacco smoking^11^. The anti-smoking law has twenty articles based on the WHO Framework Convention on Tobacco Control (FCTC)^11^. Articles of the law cover the cultivation and manufacture of tobacco, sale of tobacco, secondhand smoking, and others. By law, smoking is prohibited in public places such as governmental institutions and public workplaces. Also, the law states that sales of tobacco and its derivatives are prohibited to minors. Tobacco advertisements, promotions, and sponsorship are banned in Saudi Arabia. The anti-smoking law also encourages governmental and private entities to issue fines for violating the law. Penalties differ based on the violation; for example, the fine for violating the cultivation and manufacturing tobacco Article is SAR 20000 (100 Saudi Arabian Riyals about 27 US$), while the fine for smoking in smoke-free areas listed in Article 7 is SAR 200.

Furthermore, the Saudi healthcare delivery system provides free-of-charge smoking cessation services and a telephone quitline to provide over the phone assistance and consultation for smokers seeking quit assistance^12^. The Saudi Ministry of Health (MOH) has several tobacco cessation centers distributed across the nation to run smoking cessation programs and treatments^12^. The smoking cessation programs in Saudi Arabia offer strategies ranging from medical treatment to behavioral interventions. Medical interventions include offering consultations and prescribing medications such as nicotine replacement therapy, nicotinic receptor agonists, Varenicline, and antidepressants^12^. Behavioral interventions to treat tobacco dependence involve educational programs on the importance of smoking cessation and psychological support for tobacco smokers provided by healthcare professionals^12^.

The effectiveness of all these smoking cessation programs relies on knowing the factors that influence smokers. Factors like sociodemographic, psychological, and environmental factors play an essential role in the desire to quit smoking^12,13^. For instance, smokers with higher self-efficacy, social support, and access to smoking cessation medications have higher odds of attempting to quit^12,14^. The social cognitive theory could explain the desire to quit smoking^15^. In particular, individuals’ expected outcomes regarding smoking or quitting behavior are related to the cognitive operations of tobacco uptake or quitting^15^.

In the Saudi context, few studies have examined the desire to quit smoking^16-18^. Aljuaid et al.^18^ examined the relationship between the desire to quit smoking and the taxation policy among males only in Riyadh, Saudi Arabia; they found a significant association between the desire to quit smoking and the implementation of taxation policy. The assessment of intention to quit smoking among smoking students in Saudi Arabia revealed that intention to quit was associated with knowledge about the dangerous effects of tobacco and attitude toward banning smoking in public places^17^.

Another study focused on women attending smoking cessation clinics and found that strengthening the participants’ abstinence self-efficacy skills could help enhance smoking cessation efforts^16^. However, all these studies were limited in scope and only focused on specific populations classified based on age, gender, and region. This means that the results may not be generalizable to the country’s broader population who smoke tobacco.

The lack of literature on the desire to quit among smokers in Saudi Arabia suggests a need for more research in this area. To address these gaps in the literature, our study examines the influence of selected factors on the desire to quit among smoking adults in Saudi Arabia and investigates whether using alternative tobacco products such as e-cigarettes is associated with the desire to quit smoking.

## METHODS

### Data sources

This secondary dataset analysis used data from the 2019 Global Adult Tobacco Survey (GATS), which was conducted by the MOH in Saudi Arabia. GATS is a nationally representative survey utilizing a multistage geographically clustered sampling technique. GATS used a face-to-face household cross-sectional survey that collected data from non-institutionalized adults aged ≥15 years. The survey has a standardized questionnaire consisting of questions about main background characteristics, tobacco use, and data related to tobacco control measures such as being exposed to secondhand smoking, seeing tobacco ads, promotions, and sponsorship, utilizing smoking cessation, and others. The questionnaire was administered in person as an interview using electronic data collection procedures^19^.

### Study sample

The overall sample size collected by the MOH was 12800 households (response rate: 98%), while those who completed the survey were 11381 (response rate: 96.2%)^5^. This study included male and female adults aged ≥15 years. This study was limited to the participants who smoke any tobacco products, including e-cigarettes as an emerging tobacco product, either daily or occasionally. Participants who had never smoked tobacco products were excluded from this study.

### Measures

The primary outcome was the desire to quit tobacco smoking. The outcome variable was defined as the self-reported desire to quit smoking in the future. The outcome was assessed by the following question: ‘Which of the following best describes your thinking about quitting smoking?’. Participants who answered: ‘Quit within the next month’, ‘Thinking within the next 12 months’, or ‘Quit someday, but not next 12 months’, were recoded as ‘Desire to quit smoking’. Participants who responded: ‘Not interested in quitting’, were recoded as ‘Do not have the desire to quit smoking’. Several independent variables were examined in the study, including sociodemographic characteristics such as age (15–24, 25–34, 35–44, or ≥45 years), sex (male or female), marital status (not married or married), education (no formal education, middle school and lower, high school or equivalent degree, or college or higher), employment status (government, private, self-employed, student, housewife, or unemployed), and residency (rural or urban); awareness of smoking cessation clinics (SCCs) (not heard about SCCs or heard about SCCs), attitude toward tobacco control measures such as tobacco taxes (negative or positive), rule of smoking inside the home (not allowed or allowed), use of alternative tobacco products including conventional cigarettes, waterpipe, and e-cigarettes (yes or no). The Supplementary file gives the codebook of the variables included in this study.

### Statistical analysis

**Figure 1:**
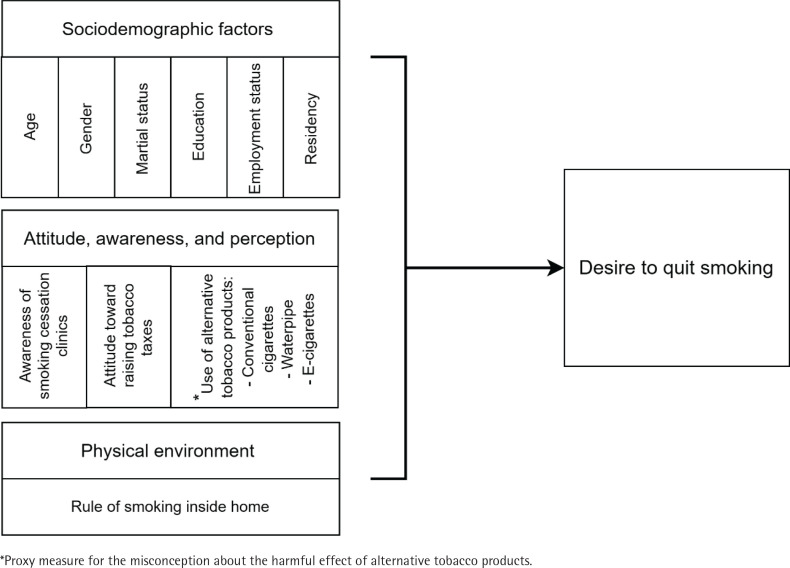
Conceptual model guides the study analysis and demonstrates factors influencing the desire to quit smoking among adult smokers using GATS Saudi Arabia, 2019

We investigated whether independent factors considered in the study were associated with the desirability to smoking cessation among adult smokers in Saudi Arabia (Figure 1). Descriptive statistics were conducted to examine the distribution of tobacco smokers by demographic characteristics. Then, bivariate analysis was utilized to explore the relationship between the desire to quit smoking and the independent variables included. Finally, logistic regression modeling was conducted to determine whether the conceptualized factors predict the desire to quit smoking. Both bivariate and multivariate logistic regression were performed to ensure consistency of the association’s magnitude between the outcome and predictors when tested separately and in the full model, holding all other variables constant. The association was reported as crude odds ratio (OR) and adjusted odds ratio (AOR) with 95% confidence intervals (CIs). The study analyses were weighted to account for the complex sampling used in the GATS. The significance of statistical analysis was set at p<0.05. The analyses were performed using STATA version 17.

## RESULTS

A total of 11381 individuals participated in and completed the GATS-Saudi Arabia questionnaire in 2019. Of the total sample, 1667 participants (14.6%) were tobacco smokers who were included in the study. The majority of the tobacco smokers were males (91.3%), 66.4% of them were married, and more than two-thirds were residing in urban areas. Approximately 36% of tobacco smokers were aged 25–34 years, 46.4% of the sample had completed a high school or equivalent degree, and 40.1% of them were working in the government sector. Furthermore, 60% of tobacco smokers were aware of SCCs, and about 85% supported raising tobacco taxes and had strict rules against smoking inside their homes.

The most tobacco products used were cigarettes (89%), followed by waterpipe (30%). Only 3.2% of tobacco users in the study sample used e-cigarettes. The majority of the tobacco smokers (82.4%) were interested in quitting smoking. Table 1 presents the distribution of tobacco users by the study’s dependent and independent variables.

**Table 1:** Distribution of tobacco smokers by the dependent and independent variables, GATS Saudi Arabia, 2019 (N=1667)

*Characteristics*	*n^a^*	*Unweighted %*	*Weighted %*
**Gender**			
Female	145	8.7	8.3
Male	1522	91.3	91.8
**Age** (years)			
15–24	313	18.8	11.9
25–34	602	36.1	36.9
35–44	493	29.6	24.7
≥45	259	15.5	26.6
**Education level**			
No formal education	140	8.4	11.1
Middle school and lower	241	14.5	15.5
High school or equivalent degree	770	46.4	43.2
College or higher	510	30.7	30.3
**Marital status**			
Not married	560	33.7	35.3
Married	1104	66.4	64.7
**Employment status**			
Government	663	40.1	36.4
Private	340	20.6	24.9
Self-employed	187	11.3	13.2
Student	175	10.6	7.5
Housewife	72	4.4	3.1
Unemployed	216	13.1	14.8
**Residence**			
Rural	521	31.3	14.5
Urban	1146	68.8	85.5
**Awareness of SCC**			
No	661	40.0	41.0
Yes	985	60.0	59.0
**Attitude toward raising tobacco taxes**			
Negative	218	14.3	18.0
Positive	1311	85.1	82.0
**Use of tobacco products**			
**Cigarettes**			
No	183	11.0	11.2
Yes	1484	89.0	88.8
**Waterpipe**			
No	1165	70.0	62.0
Yes	502	30.0	38.0
**E-cigarettes**			
No	1581	96.8	96.5
Yes	53	3.2	3.5
**Smoking inside the home**			
Not allowed	218	14.3	18.0
Allowed	1311	85.7	82.0
**Desire to quit smoking**			
No	232	17.6	19.5
Yes	1084	82.4	80.5

a n: unweighted number of participants.

Table 2 shows the associations between the characteristics of the study sample and the desire to quit smoking. Of the total sample, the percentage of cigarette smokers who were interested in quitting (58%) was higher than that of waterpipe users who were interested in quitting smoking (17.1%). The factors, awareness of SCCs, attitude toward raising tobacco taxes, and rule of smoking inside the home had significant associations with the desire to quit smoking. Tobacco smokers who had awareness of SCCs in their regions (p<0.001), attitude toward raising tobacco taxes (p=0.002), and rule of smoking inside the home (p=0.023) were more likely to have the desire to quit smoking compared to their counterparts. The other factors did not show significant associations with the desire to quit smoking. Aligned with bivariate analysis, the multivariate logistic regression analysis showed that tobacco smokers who had heard of SCCs (AOR=3; 95% CI: 1.8–5), supported the raise of tobacco taxes (AOR=2.3; 95% CI: 1.4–3.8), and had a strict rule of smoking inside the home (AOR=2; 95% CI: 1.1–3.9) were positively associated with the desire to quit smoking. Other variables showed no association with the desire to quit smoking (Table 3).

**Table 2:** Bivariate analysis of factors associated with desire to quit smoking among smokers, GATS Saudi Arabia, 2019 (N=1667)

*Characteristics*	*Desire to quit smoking*		
	*No*	*Yes*	*p*
	*n^a^ (Weighted %)*		
**Gender**			0.205
Female	29 (27)	75 (73)	
Male	203 (19)	1009 (81)	
**Age** (years)			0.433
15–24	38 (21)	211 (79)	
25–34	84 (23)	386 (77)	
35–44	71 (16)	316 (84)	
≥45	39 (18)	171 (82)	
**Education level**			0.222
No formal education	25 (13)	82 (87)	
Middle school and lower	36 (25)	151 (75)	
High school or equivalent degree	105 (21)	491 (79)	
College or higher	65 (16)	356 (84)	
**Marital status**			0.140
Not married	87 (23)	355 (77)	
Married	145 (18)	728 (82)	
**Employment status**			0.866
Government	88 (19)	438 (81)	
Private	47 (20)	227 (80)	
Self-employed	24 (17)	120 (83)	
Student	23 (20)	116 (80)	
Housewife	15 (30)	32 (70)	
Unemployed	32 (18)	147 (82)	
**Residence**			0.223
Rural	74 (15)	330 (85)	
Urban	158 (20)	754 (80)	
**Awareness of SCC**			<0.001***
No	125 (27)	386 (83)	
Yes	105 (14)	697 (86)	
**Attitude toward raising tobacco taxes**			0.002**
Negative	120 (25)	363 (75)	
Positive	104 (14)	682 (86)	
**Use of tobacco products Cigarettes**			0.541
No	28 (17)	115 (83)	
Yes	204 (20)	969 (80)	
**Waterpipe**			0.955
No	151 (20)	799 (80)	
Yes	81 (20)	285 (80)	
**E-cigarettes**			0.546
No	222 (20)	1040 (80)	
Yes	8 (14)	35 (86)	
**Smoking inside the home**			0.023*
Not allowed	51 (28)	129 (72)	
Allowed	155 (17)	915 (83)	

a n: unweighted number of participants.

* p<0.05,

** p<0.01,

*** p<0.001.

**Table 3:** Odd ratios of factors associated with desire to quit smoking among smokers, GATS Saudi Arabia, 2019 (N=1667)

*Characteristics*	*Desire to quit smoking*			
	*Bivariate logistic regression*		Multivariate logistic regression*	
	*OR*	*95% CI*	*AOR*	*95% CI*
**Gender**				
Female (Ref.)			1	
Male	1.6	0.8–3.2	1.9	0.7–5.4
**Age** (years)				
15–24 (Ref.)			1	
25–34	0.9	0.5–1.7	0.7	0.4–1.5
35–44	1.4	0.7–2.7	1.4	0.6–3.2
≥45	1.2	0.6–2.3	1.1	0.4–2.7
**Education level**				
No formal education (Ref.)			1	
Middle school and lower	0.4	0.2–1.1	0.4	0.2–1.3
High school or equivalent degree	0.5	0.2–1.2	0.5	0.2–1.4
College or higher	0.7	0.3–1.8	0.7	0.2–1.9
**Marital status**				
Not married (Ref.)			1	
Married	1.4	0.9–2.3	1.2	0.6–2.1
**Employment status**				
Government (Ref.)			1	
Private	0.9	0.5–1.7	1.4	0.7–2.8
Self-employed	1.2	0.5–2.6	2.3	0.9–5.8
Student	0.9	0.4–2.1	1.3	0.6–3.2
Housewife	0.6	0.2–1.6	1.2	0.3–5.5
Unemployed	1.1	0.5–2.2	1.7	0.8–3.5
**Residence**				
Rural (Ref.)			1	
Urban	0.7	0.4–1.2	0.8	0.4–1.3
**Awareness of SCC**				
No (Ref.)			1	
Yes	2.3	1.4–3.7	3	1.8–5.0
**Attitude toward raising tobacco taxes**				
Negative (Ref.)			1	
Positive	2.1	1.3–3.3	2.3	1.4–3.8
**Use of tobacco products**				
**Cigarettes**				
No (Ref.)			1	
Yes	0.8	0.4–1.6	1.1	0.5–2.8
**Waterpipe**				
No (Ref.)			1	
Yes	1.0	0.6–1.6	1.3	0.5–2.8
**E-cigarettes**				
No (Ref.)				
Yes	1.5	0.4–5.1	-	-
**Rule of smoking inside the home**				
Not allowed (Ref.)			1	
Allowed	1.9	1.1–3.3	2	1.1–3.9

* Association between the outcome variable and the independent variable was estimated, holding all other variables constant.

AOR: adjusted odds ratio. Ref: reference.

## DISCUSSION

This study is one of the first to assess the desire to quit smoking among Saudi tobacco smokers from the recent GATS in Saudi Arabia. The study findings revealed that the national prevalence of tobacco smoking was 14.6%. The sociodemographic characteristics of tobacco smokers were mostly males (91.3%), married (66.4%), urban residents (68.8%), aged 25–34 years (36.1%), completed high school (46.4%), and employed in government sectors (40.1%). Furthermore, tobacco smokers who were aware of SCCs, supported the increase of taxes on tobacco products, and applied strict rules of smoking inside the home, were more likely to have a greater desire to quit tobacco smoking.

The sociodemographic characteristics of tobacco smokers in this study were consistent with several recent studies which assessed tobacco smoking in Saudi Arabia. Tobacco was dominantly smoked by males, married, educated, and governmentally employed individuals^20^. These findings were similar across all tobacco products in the country^2,20,21^. The significant difference seen between this study and others was the age of tobacco users, as our findings revealed that most of the individuals were aged 25– 34 years, whereas others reported cigarette smoking explicitly among older individuals^2,20,21^. Of note, these studies were not nationally representative of tobacco use in the country.

There are different forms of tobacco products in Saudi Arabia, such as cigarettes, waterpipe, and e-cigarettes, as emerging tobacco products in recent years^5^. Our findings showed that the desire to quit smoking varies based on the use patterns of tobacco products. According to the data, the higher percentage of smokers who were interested in quitting were cigarette users (58%) followed by waterpipe users (17.1%) and e-cigarette users (2.1%) (Table 2). This finding could be explained by the misconception about the harmful effects of waterpipe and e-cigarettes^22^. In regard to waterpipe use, the lack of interest in quitting the waterpipe may be related to the cultural identity and the sense of social cohesion that the waterpipe brings to its users, as most waterpipe users smoke with their friends and relatives^22^.

With respect to e-cigarette, its use was widely reported in the literature as a method to quit tobacco^23,24^. A cross-sectional survey of 3374 participants in Saudi Arabia revealed that 19.5% used e-cigarettes to quit cigarette smoking, and 16.2% used them as a safer alternative to cigarette smoking^25^. Surveying 1404 smokers in Saudi Arabia revealed that approximately 70% believed that e-cigarettes would help them quit smoking^26^. Although there was no statistical association between the desire to quit smoking and the use of alternative tobacco products, the finding urges further assessment of the barriers that hinder tobacco quitting, such as nicotine dependence. In addition, those who used e-cigarettes, for example, may hold positive expectations about the safety of e-cigarette use. All of these urge further investigations to uncover the reasons behind the continuity of tobacco use, in particular, e-cigarettes and discover interventions that enhance the desire to quit among tobacco smokers.

Tobacco smoking is a chronic relapsing disorder, and smokers are expected to have several quit attempts to sustain abstinence from tobacco smoking successfully^27^. Those with a greater desire to quit tobacco smoking are more likely to have greater quit attempts which necessitate the need to motivate smokers to keep trying to quit tobacco smoking^27^. Our findings revealed that tobacco smokers who were aware of the SCCs, supported raising tobacco taxes, and had a smoke-free rule at home, had a greater desire to quit tobacco smoking. This notion is partially supported by two recent national studies related to tax policy implementation on tobacco products in Saudi Arabia. First, taxation on tobacco products effectively decreased the affordability and consumption of cigarette smoking^28^. Second, the strategies adopted by tobacco smokers who attended SCCs after implementing taxation on tobacco cigarettes mainly were decreasing the number of cigarettes smoked and attempting to quit tobacco cigarettes^29^. Such findings indicate the importance of tobacco control interventions, such as taxes on tobacco products, implementing smoke-free policies in various places and providing smoking cessation services, in affecting smoking behavior and promoting cessation among tobacco smokers.

### Limitations

The study has identified several important factors associated with the desire to quit smoking. However, it is important to interpret the findings cautiously, given some limitations of the study design. In particular, using a household survey may have introduced response bias since it relies on self-reporting. Participants may not accurately report their tobacco use due to social desirability bias or lack of awareness of their own behavior. A potential limitation of our study is the possibility of reverse causality in the association between awareness of SCCs and the desire to quit smoking, as the desire to quit smoking among smokers may precede the awareness of available SCCs. Another limitation of our study is the lack of information about smoking quit among participants. While our analysis identified several factors associated with the desire to quit smoking, we were unable to assess the actual impact of these factors on quitting behavior. Furthermore, due to missing data, we were unable to include information on the frequency, duration, and intensity of tobacco use in our analysis. This missing information could have strengthened our analysis and identified more predictors. Also, the small sample size of e-cigarette users (3.2%) prevented us from providing an estimate of the association (Table 3). Finally, it is important to note that our study used a cross-sectional design. This type of study is limited in its nature in terms of the ability to establish causal relationships, as it only collects data from participants at a single point in time.

## CONCLUSIONS

Tobacco use is a global public health problem. The optimal solution to address this issue is to prevent tobacco use and encourage tobacco users to quit. This study is the first to use nationally representative data to investigate the barriers and facilitators that influence smoking cessation. The study provides valuable insights into factors associated with the desire to quit smoking among Saudi smokers. Our findings have identified important factors that could inform the development of more effective policy interventions targeting smokers in Saudi Arabia. Further research is needed to identify other factors that may foster the desire to quit smoking and increase successful quitting among smokers in Saudi Arabia.

## Data Availability

The data supporting this research are available from the authors on reasonable request.
