# Supplementary material for: Factors associated with the desire to quit tobacco smoking in Saudi Arabia: Evidence from the 2019 Global Adult Tobacco Survey
Source: Tob Induc Dis. 2023 Mar 3;21:33. doi: 10.18332/tid/159735 (PMC9983308; doi:10.18332/tid/159735)
Supplement: Supplementary file 1 [file TID-21-33-s1.pdf]

Appendix 1. Codebook for included variables

**Variables**	**Recode Response Options**
**Desire to quit smoking**<br>Which of the following best describes your thinking about quitting smoking?	1 = No (not interested in quitting)<br>2 = Yes (quit within the next month, thinking within the next 12 months, quit someday, but not next 12 months)
**Gender**<br>Is this person male or female?	1 = Male<br>2 = Female
**Age**<br>How old are you?	1 = 15-24<br>2 = 25-34<br>3 = 35-44<br>4 = 45 +
**Marital status**<br>What is your marital status?	1 = Not married (single, separated, divorced, widowed)<br>2 = Married (married)
**Education**<br>What is the highest level of education you have completed?	1 = No formal education (no formal schooling)<br>2 = Middle school and less (less than primary school completed, primary school completed, less than secondary school completed, secondary school completed)<br>3 = High school or equivalent degree (high school completed)<br>4 = College or higher education (college/university completed, post graduate degree completed)

**Employment status**<br><br>Which of the following best describes your main work status over the past 12 months?	1 = Government (government employee)<br>2 = Private (non-government employee)<br>3 = Self-employed (self-employed)<br>4 = Student (student)<br>5 = Housewife (homemaker)<br>6 = Unemployed (retired, unemployed, able to work, unemployed, unable to work)
**Residency***	1 = Rural<br>2 = Urban
**Use of alternative tobacco products**<br><br>*Cigarettes:*<br><br>Do you currently smoke tobacco on a daily basis, less than daily, or not at all?<br><br>*Waterpipe:*<br>Do you currently smoke waterpipe on a daily basis, less than daily, or not at all?<br><br>*E-cigarettes*<br>Do you currently use electronic cigarettes on a daily basis, less than daily, or not at all?	1 = No (not at all)<br>2 = Yes (daily, less than daily)
**Rule of smoking inside the home**<br><br>Which of the following best describes the rules about smoking inside of your home: Smoking is allowed inside of your home, smoking is generally not allowed inside of your home but there are exceptions, smoking is never allowed inside of your home, or there are no rules about smoking in your home?	1 = Not allowed (never allowed)<br>2 = Allowed (allowed, not allowed but exceptions, no rules)
**Awareness of smoking cessation clinics**<br><br>Have you ever heard of a smoking cessation clinic (in your region) that offers help to quit smoking?	1 = No<br>2 = Yes

**Attitude toward raising tobacco taxes**<br><br>Would you favor or oppose increasing taxes on tobacco products?	1 = Negative attitude (oppose)<br>2 = Positive attitude (favor)

Note: (*) No specific question was asked to the participants. Value was assigned based on the geographical clusters of households included in the study.

© 2023 Monshi S.S. et al.
